# Electrolytic Cleaning as Part of Surgical Reconstructive Peri-Implantitis Treatment: A Case Series

**DOI:** 10.3390/dj13060237

**Published:** 2025-05-26

**Authors:** Jarno Hakkers, Henny J. A. Meijer, Yvonne C. M. de Waal, Gerry M. Raghoebar

**Affiliations:** 1 Department of Oral and Maxillofacial Surgery, University Medical Center Groningen, University of Groningen, 9713 GZ Groningen, The Netherlands; h.j.a.meijer@umcg.nl (H.J.A.M.);; 2 Department of Periodontology, Center for Dentistry and Oral Hygiene, University Medical Center Groningen, University of Groningen, 9713 GZ Groningen, The Netherlands; y.c.m.de.waal@umcg.nl; 3 Department of Restorative Dentistry, Center for Dentistry and Oral Hygiene, University Medical Center Groningen, University of Groningen, 9713 GZ Groningen, The Netherlands

**Keywords:** peri-implantitis, dental implant, surgical treatment, electrolytic cleaning

## Abstract

**Background/Objectives:** The aim of this study was to assess the workflow and evaluate the clinical, radiographic and patient-reported outcome measures of surgical reconstructive peri-implantitis treatment aided with an electrolytic cleaning device. **Methods:** This case series describes three patients that presented with peri-implantitis surrounding an implant in the anterior maxilla, eligible for surgical reconstructive peri-implantitis treatment. The implant crown was removed, and the patients were surgically treated during which an electrolytic cleaning device was used to clean the implant surface. Thereafter, the implant site was augmented using locally harvested bone and a bovine bone substitute. After 6 months of submerged healing, the implant crown was replaced. Follow-up visits were performed 3 and 6 months after restoration placement. Clinical and radiographical parameters, as well as Patient-Reported Outcome Measures (PROMs) and Pink Esthetic Scores (PESs) were collected. **Results:** The data collected indicate a positive trend with regard to peri-implant pocket depth, bleeding and suppuration scores, as well as the peri-implant marginal bone level for the evaluated cases six months postoperatively. PROMs with regard to pain experience varied between 4.5 and 5.9 on the Visual Analogue Scale (VAS). After 12 months, the PES increased in two cases and decreased in one case. **Conclusions:** This case series provides a comprehensive overview of the surgical reconstructive peri-implantitis treatment using an electrolytic cleaning device, highlighting promising results with regard to the aforementioned parameters.

## 1. Introduction

Peri-implantitis is characterized by mucosal inflammation, soft tissue degradation and progressive bone loss around implants and threatens the long-term success of dental implants [1]. Addressing this condition demands effective treatment strategies that not only target microbial biofilms but also promote tissue healing and, if possible, re-osseointegration. Surgical peri-implantitis treatment could aid in achieving this [2]. However, it seems that achieving complete disease resolution is difficult [3,4].

Surgical reconstructive peri-implantitis treatment has been evaluated in randomized controlled trials [5,6]. However, the results with regard to long-term treatment success remain inconclusive. In the systematic review performed by Donos [7], it was concluded that reconstructive surgical peri-implantitis treatment does not significantly improve peri-implant clinical parameters, such as the peri-implant probing depth and bleeding score, better than access flap surgery. It must be noted that the clinical trials incorporated in the analysis present a multitude of implant decontamination protocols, predominantly involving mechanical and/or chemical decontamination strategies [7]. Given the fact that most implants placed today have a rough surface to promote osseointegration, it could be hypothesized that these treatment modalities are unable to penetrate the rough surface texture. Such conditions might allow residual biofilm to reinstate tissue inflammation [8,9,10]. Electrolytic cleaning might provide a solution to this problem [11].

In electrolytic cleaning, the implant itself is used to aid in the decontamination procedure [12]. A device is mounted on the implant, supplying the necessary voltage and current and delivering an electrolyte solution through a spray head. Under the influence of the electrical current, water molecules are split into hydrogen anions and cations. The cations are said to permeate the biofilm and extract electrons from the implant, allowing hydrogen bubbles to form that aid in dislodging the biofilm from the implant’s surface. In this sense, the entire implant surface, along with the crevices in the surface texture, is cleaned. Theoretically, this could aid in achieving an implant surface state conducive to re-establishing osseointegration. In vitro comparison of electrolytic cleaning in relation to other implant surface decontamination protocols, such as an erythritol-chlorhexidine air polishing system, show similar performances in eliminating *P. aeruginosa* biofilm from dental implants [13].

The clinical evidence supporting the use of an electrolytic cleaning device as an adjunct to surgical reconstructive surgical peri-implantitis treatment is limited. Promising results with regard to disease resolution have been demonstrated when electrolytic cleaning was compared to implant surface decontamination using hydrogen peroxide [14]. To analyze its workflow and efficacy, this study aimed at providing a clinical and radiographical insight into the use of electrolytic cleaning by evaluating three consecutive peri-implantitis patients.

## 2. Study Design

This case series evaluated the workflow and clinical treatment outcomes of the use of an electrolytic cleaning device (GalvoSurge^®^; GalvoSurge, Widnau, Switzerland) during surgical reconstructive peri-implantitis treatment by analyzing three consecutively recruited patients. Patients that were referred to the Department of Oral and Maxillofacial Surgery at the University Medical Center Groningen (UMCG), The Netherlands, were checked for eligibility to participate in the study. The research protocol was approved by the Medical Ethics Review Board of the UMCG (METc nr. 2021/732). This study was written in accordance with the CARE guidelines for case reports [15].

## 3. Patient Information

Consecutive patients diagnosed with peri-implantitis affecting an implant in the anterior maxilla (cuspid to cuspid region) underwent a preliminary intake for eligibility assessment. Definitive inclusion was confirmed upon identifying a three- or four-wall peri-implant bone defect during surgery. Peri-implantitis was diagnosed in accordance with the criteria as stated by the “2017 World Workshop on the Classification of Periodontal and Peri-Implant Diseases and Conditions” [1]: Presence of bleeding and/or suppuration on gentle probing, increased probing depth compared to previous examinations and the presence of bone loss beyond crestal bone level changes resulting from initial bone remodeling. In the absence of previous examination data, peri-implantitis was defined as the presence of bleeding and/or suppuration on gentle probing (BoP/SoP), probing depths (PDs) of ≥ 6 mm and bone levels ≥ 3 mm apical of the most coronal portion of the intraosseous part of the implant. The exclusion criteria were as follows: medical/general contra-indications for the procedure, a history of local radiotherapy to the head and neck region, pregnancy and/or lactation, uncontrolled diabetes mellitus (HbA1c < 7% or < 53 mmol/mol), the use of intravenous bisphosphonates, known allergy to chlorhexidine or amoxicillin, significant contra-indications to the use of antibiotics due to other medicines, the long-term use of anti-inflammatory drugs, incapability of performing basic oral hygiene measures as a result of physical and/or mental disorders, implants with bone loss > 2/3 of implant length or previous surgical peri-implantitis treatment. Three consecutively recruited patients agreed to participate in the study. Written informed consent was obtained from all patients.

## 4. Timeline and Diagnostic Assessment

**Figure 1:**
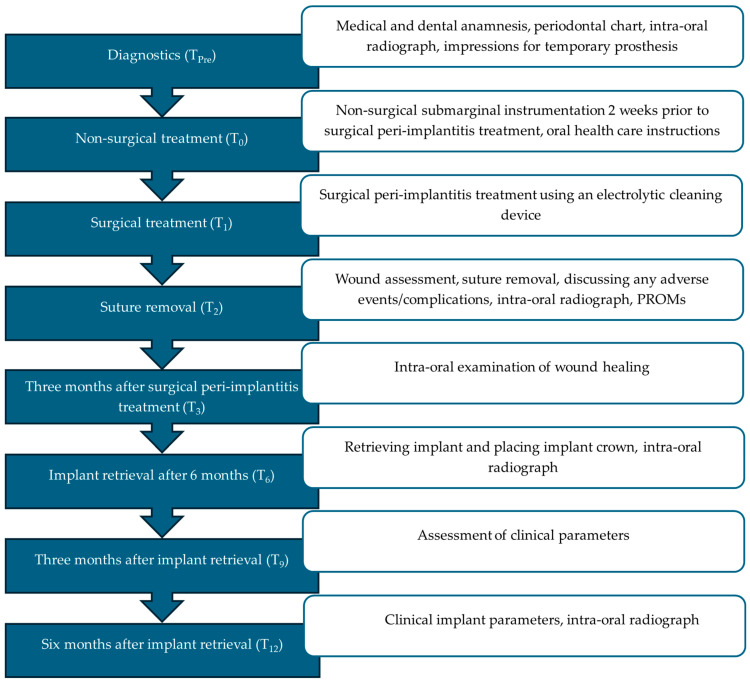
Timeline.

Patient characteristics are described in Table 1, and the study workflow is reported in Figure 1. The clinical parameters were scored at six sites per implant by one examiner (JH). The presence of plaque (%) was visually analyzed using a dental probe and scored as either present or absent. The PD (mm) was assessed in mm using a Hu-Friedy^®^ PCPUNC156 periodontal probe (Hu-Friedy Manufacturing, Chicago, IL, USA), as well as the midfacial keratinized tissue width (mm). Concomitant peri-implant bleeding and/or suppuration scores (BS, SS; %) were recorded as either present or absent. The radiographic bone level (measured from the most coronal aspect of the intraosseous segment of the implant) was measured using DICOM software (DicomWorks, Biomedical Engineering, UMCG, Groningen, The Netherlands) [4]. Patient-Related Outcome Measures (PROMs) were surveyed by means of an Adverse Events questionnaire pertaining to, e.g., pain (Visual Analogue Scale (VAS)) and the use of pain medication. Esthetic results were evaluated using the Pink Esthetic Score (PES) [16]. The scoring was executed by one examiner (HM).

## 5. Therapeutic Intervention

### 5.1. Patient Diagnostics

**Figure 2:**
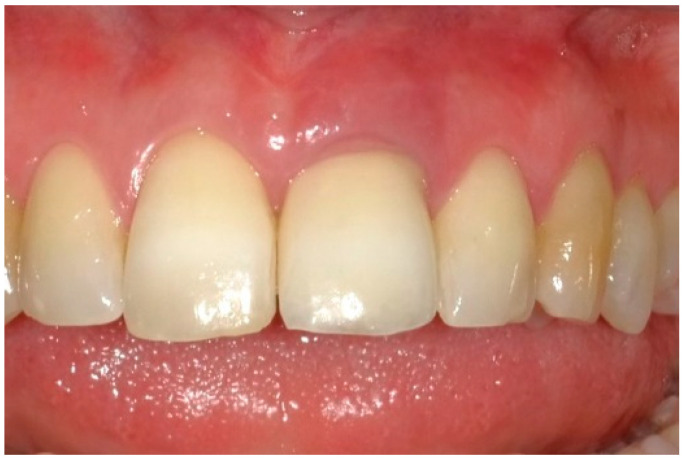
Baseline status of the peri-implant mucosa.

Case 1 presents with an implant in position 22. Radiographical assessment reveals that the apex of the implant is strongly inclined mesially. The patient reports a history of nocturnal bruxism and previously used a splint, which no longer fits. Case 2 presents with an implant in position 12, for which no implant- or prosthesis-related factors were identified that could relate to the onset of the peri-implant inflammation. The patient reports no nocturnal bruxism and does not wear a splint. The case elaborated on in this study represents Case 3. During the initial appointment (T_Pre_, Figure 2), data pertaining to the patient’s dental and medical history were gathered, alongside specifics regarding the type of implant utilized (Table 1). Subsequently, a periodontal chart was completed. An intraoral radiograph of the implant was also captured. To ensure consistency in intraoral radiographs and to maintain perpendicularity, a custom X-ray holder and paralleling technique were utilized. To assess changes in the mid-facial mucosal level over time, the gingival position on the midbuccal side of the implant crown was measured. To establish a consistent height reference, a local putty impression was made, which was trimmed to approximately the middle of the implant crown. (Elite HD + Putty Soft Regular, Zhermack SpA, Badia Polesine, Italy) [4].

### 5.2. Non-Surgical Pre-Treatment

The surgical intervention (T_1_) followed a preoperative non-surgical treatment intervention (T_0_) conducted 1–2 weeks prior to surgery, aimed at reducing peri-implant inflammation during the surgical procedure. Two experienced dental hygienists carried out non-surgical submarginal peri-implant instrumentation employing an air-polishing device (PerioFlow^®^; EMS, Nyon, Switzerland). Oral self-care guidelines were provided, advocating the use of an electric toothbrush and interdental brushes. None of the cases presented have been diagnosed with periodontitis.

### 5.3. Surgical Treatment

**Figure 3:**
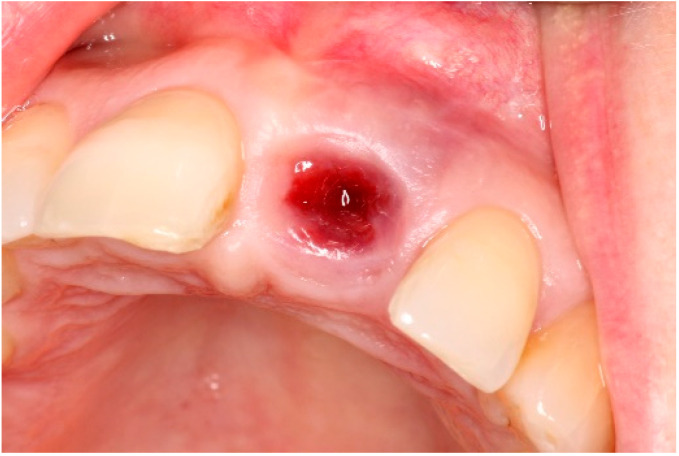
Crown removal pre-operatively.

**Figure 4:**
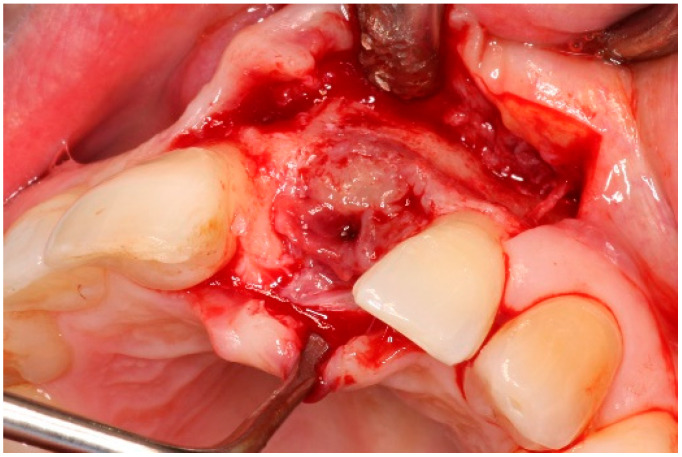
Flap reflection, exposure of the implant surface.

**Figure 5:**
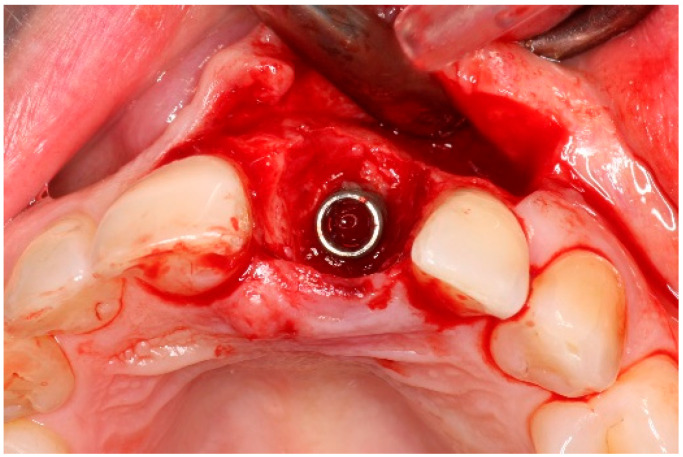
Granulation tissue removal.

**Figure 6:**
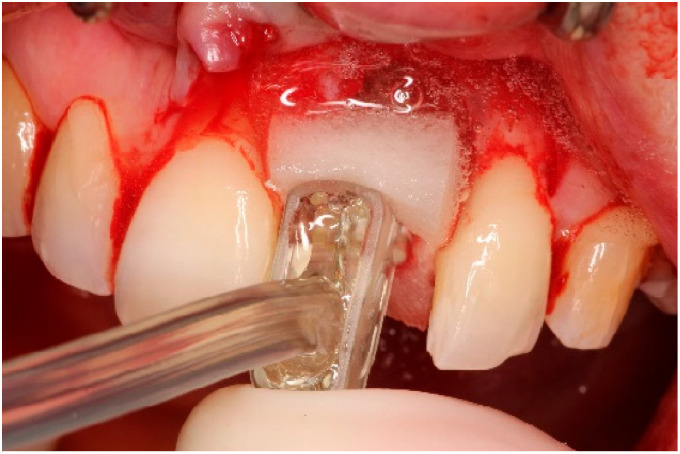
Surface decontamination using electrolytic cleaning.

**Figure 7:**
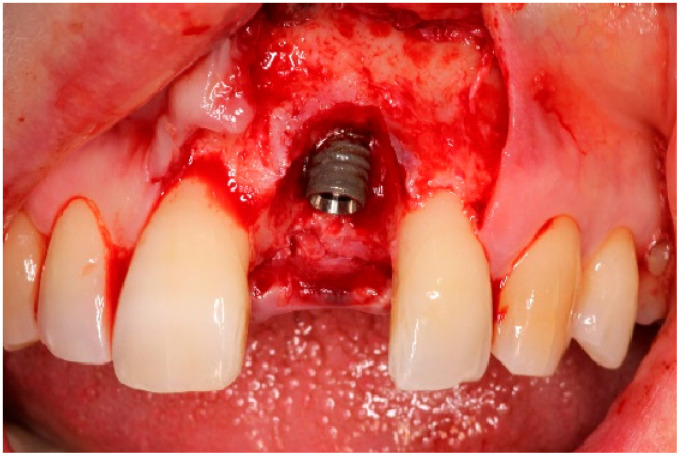
Implant status directly after electrolytic cleaning.

**Figure 8:**
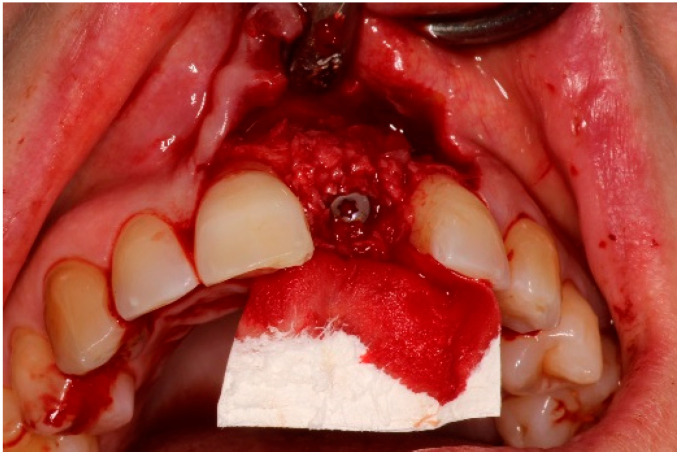
Application of bone substitute and collagen membrane.

**Figure 9:**
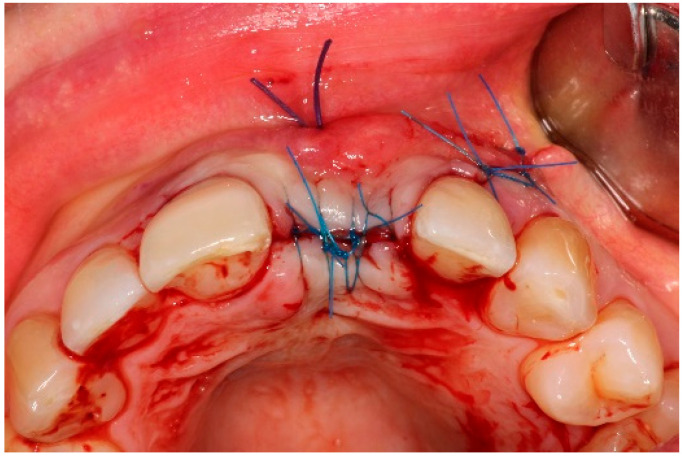
Surgery site directly after suturing.

Two weeks after non-surgical treatment, the patients were surgically treated. All surgical treatments were performed by one experienced oral and maxillofacial surgeon (GM). After removal of the implant crown (Figure 3), local anesthesia was administered. An access flap with a single distal releasing incision was raised buccally and lingually (Figure 4). Granulation tissue was removed using titanium curettes (Hu-Friedy Manufacturing, Chicago, IL, USA) (Figure 5). When the implant surface was deemed to be visually clean, the electrolytic cleaning device was used in accordance with the manufacturers’ protocol (Figure 6). After the procedure was finished (Figure 7), a cover screw was placed in the implant. In the region of the anterior nasal spine, local cortical bone was harvested using a bone scraper (SafeScraper^®^ TWIST, Geistlich Pharma, Wolhusen, Switzerland). The autologous bone was administered on the implant surface (Figure 8). Directly after administering the autologous bone to the implant surface, the augmented site was additionally covered with a bovine bone substitute (Geistlich Bio-Oss^®^, Geistlich Pharma, Wolhusen, Switzerland). The augmentation site was then protected using a collagen membrane (Geistlich Bio-Gide^®^, Geistlich Pharma, Wolhusen, Switzerland). After the collagen membrane was placed, the wound was sutured using a monofilamentous non-absorbable 5–0 suture (Ethilon™, Ethicon, Inc., Johnson & Johnson, New Brunswick, NJ, USA) (Figure 9). The site was allowed to heal submerged for six months. In the meantime, the patient received a temporary removable prosthetic, constructed in such a way that the prosthetic did not put pressure on the healing site.

## 6. Follow-Up

**Figure 10:**
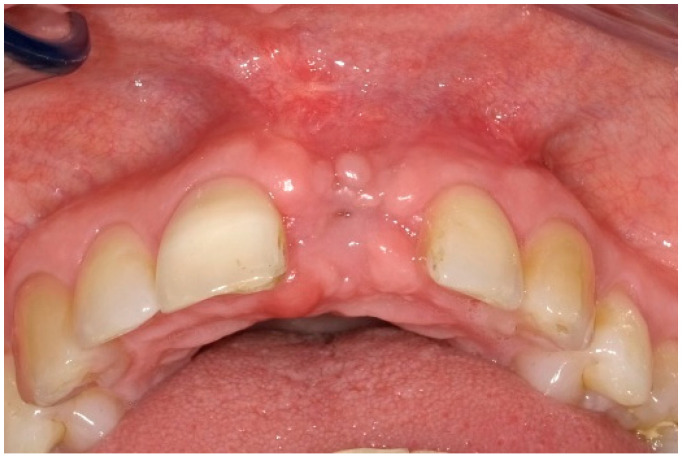
Two weeks postoperatively.

**Figure 11:**
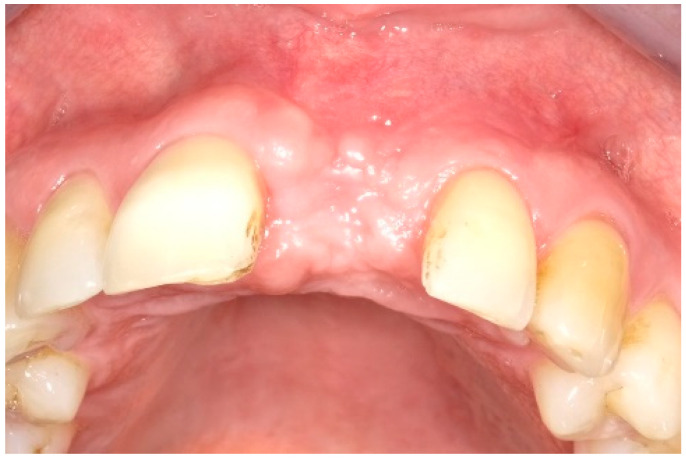
Three months postoperatively.

**Figure 12:**
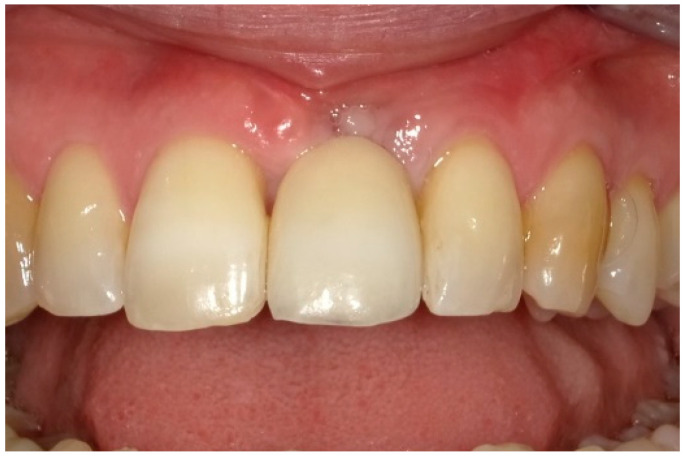
Directly after crown replacement surgery.

**Figure 13:**
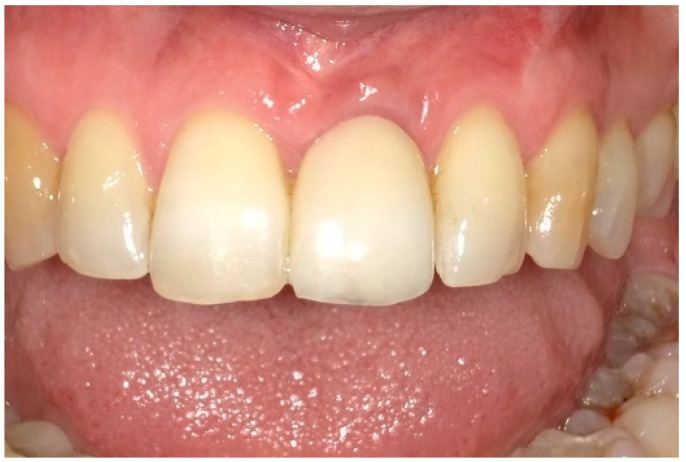
Six months after crown replacement.

**Figure 14:**
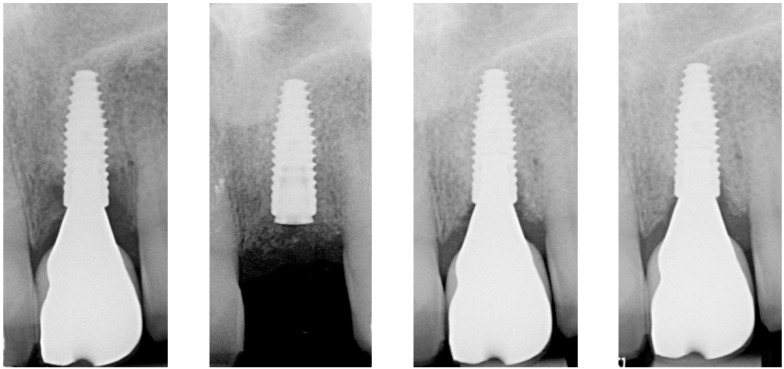
Peri-implant bone level over time (from **left** to **right**: T_Pre_, T_2_, T_6_, T_12_).

**Figure 15:**
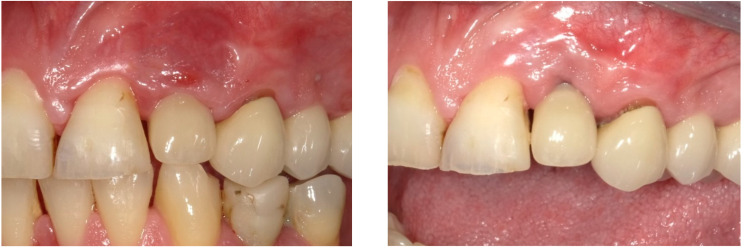
Baseline (T_Pre_, (**left**)) and postoperative (T_12_, (**right**)) clinical situation, Case 1.

**Figure 16:**
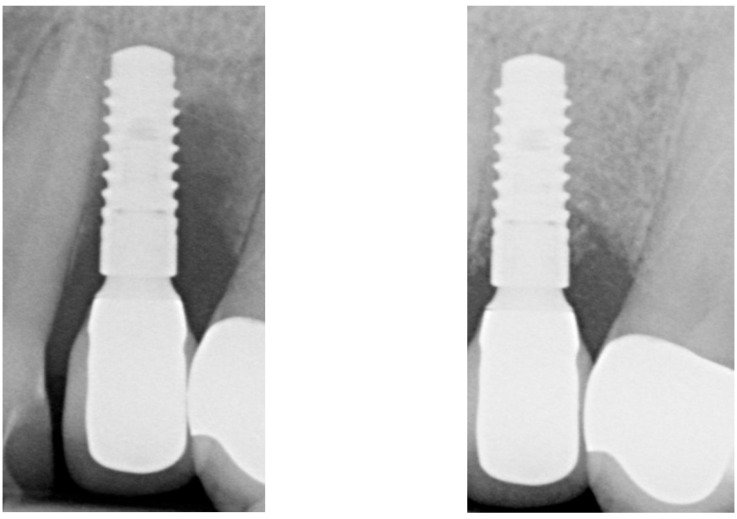
Baseline (T_Pre_, (**left**)) and postoperative (T_12_, (**right**)) radiographical situation, Case 1.

**Figure 17:**
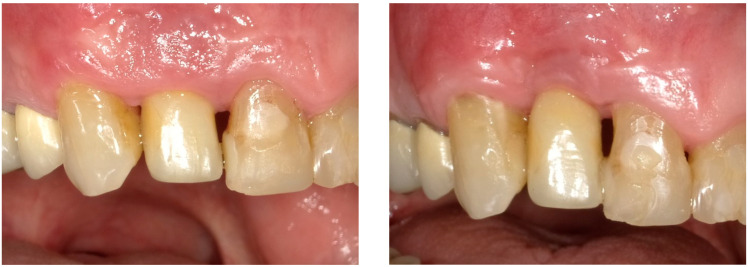
Baseline (T_Pre_, (**left**)) and postoperative (T_12_, (**right**)) clinical situation, Case 2.

**Figure 18:**
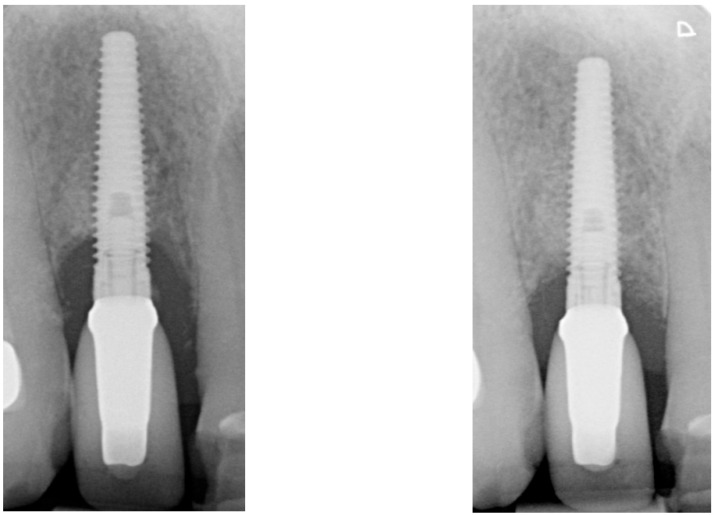
Baseline (T_Pre_, (**left**)) and postoperative (T_12_, (**right**)) radiographical situation, Case 2.

After two weeks, the sutures were removed (T_2_, Figure 10). Any adverse events that had occurred were discussed, and an intraoral radiographic image was taken. Three months postoperatively (T_3_, Figure 11), the wound was clinically assessed, and an intraoral radiograph was taken in order to inspect the submerged augmentation site. After six months of submerged healing, implant retrieval was performed using local anesthetics (T_6_, Figure 12). The cover screw was removed, and the crown was repositioned on the implant. The peri-implant mucosa was allowed to heal for 3 months before clinical assessment was performed. Three (T_9_) and six months (T_12_, Figure 13) after implant retrieval, clinical parameters were recorded (PD, BS, SS, midfacial mucosal level) and an intraoral radiograph was taken (Figure 14). The clinical and radiographic data are presented in Table 2, and the PES outcomes are presented in Table 3. Furthermore, the baseline (T_pre_) and postoperative (T_12_) clinical and radiographical situations regarding Cases 1 and 2 are presented in Figure 15, Figure 16, Figure 17 and Figure 18.

## 7. Outcomes

At T_Pre_, the mean peri-implant PD for all three cases was 8.5 mm, with the deepest pockets varying between 9.0 and 11.0 mm. Concomitantly, all cases presented with a bleeding score of 100.0%. There was also suppuration on probing present in all cases. Furthermore, the largest radiographic bone level measurement per case varied between 3.2 and 7.6 mm. Analysis of the intraoral radiograph at T_2_ showed that the largest bone level measurements were between 0.0 and 3.9 mm. Based on a scale of 0–10, the VAS scores varied between 4.5 and 5.9 with pain medication being limited to the use of acetaminophen, 500–1000 mg, 2–6 times daily for 2–6 days. With regard to the clinical assessment, interim evaluation at T_3_ demonstrated that in two out of the three cases, the implant neck had become visible. At that timepoint, the peri-implant mucosa did not display signs of inflammation.

After crown repositioning at T_6_, the peri-implant radiographic bone level varied between 1.1 and 3.5 mm. The mean peri-implant PDs at T_9_ were 3.0–3.5 mm (with the deepest pockets varying between 4.0 and 6.0 mm) with a bleeding score of 16.7–100.0%. At this timepoint, suppuration scores were 0.0% in all cases. Thereafter, at T_12_, the mean peri-implant PD varied between 3.0 and 4.0 mm, the deepest pockets being 4.0–6.0 mm, with mean bleeding scores of 33.3–83.3%. Suppuration on probing was still absent in all cases. The concomitant radiographic bone level varied between 1.5 and 3.5 mm. When compared to the baseline measurements, the midfacial mucosal recession (measured from the edge of the fabricated putty impression to the top of the mucosal margin) that occurred varied between 1.0 and 4.0 mm. The PES had improved at T_12_ in two cases, one case showed a marginal decrease (Table 3).

## 8. Discussion

This case series describes the workflow and follow-up outcomes regarding surgical reconstructive peri-implantitis treatment aided by electrolytic cleaning device. It contributes to the yet limited body of clinical studies on a relatively novel approach to implant surface decontamination during surgical peri-implantitis management. In the three cases that are presented, positive results can be observed when it comes to clinical and radiographic parameters.

All patients presented with deep peri-implant pockets at baseline assessment, posing notable challenges for treatment. When assessing the outcomes of surgical reconstructive peri-implantitis therapy, the existing literature indicates that peri-implant PD and marginal bone levels (MBLs) are expected to improve by approximately 2.8–3.7 mm and 1.1–2.0 mm, respectively, while mucosal recession is expected to be restricted to 0.7–1.1 mm [5,6,17]. PDs in the cases presented in this study reduced between 4.0 and 6.0 mm one year postoperatively with a 6-month submerged healing time after surgical debridement and augmentation. This exceeds the expected peri-implant PD reduction. Furthermore, radiographic MBLs increased by around 0.8–4.3 mm. None of the cases presented with peri-implant SoP at T_12_.

When facing esthetic challenges like the cases examined in this study, mucosal recession should ideally be minimized as much as possible. Midfacial mucosal recession in the presented cases varied between 1.0 and 4.0 mm. The randomized controlled trial performed by Derks [6] showed that, after surgical reconstructive peri-implantitis treatment, mucosal recession occurred up to 3.0 mm. It therefore seems that electrolytic cleaning would obtain similar results. However, this should be verified in larger prospective studies. While cases presenting with three- and four-wall defects were selected for this study, it should be noted that some cases presented with a combined horizontal and vertical bone defect. Horizontal bone defects exhibit reduced capacity for augmentation when compared to vertical bone defects, which could account for the observed increase in mucosal recession [18,19]. This underlines the importance of careful case selection and the anticipation of mucosal recession when horizontal bone loss is present, which should also be communicated with the patient when treating esthetically sensitive cases.

While these improvements seem to be promising, none of the cases appear to meet recommended treatment outcomes as proposed by, e.g., Herrera [20] in their clinical practice guideline (≤1 point of BoP, absence of SoP, PD ≤ 5 mm and absence of progressive bone loss compared to pre-treatment bone levels). This could imply that the long-term peri-implant tissues are not stable, with the possibility of disease progression and reoccurrence of clinical inflammation and progressive bone loss. Reinstating peri-implant health after surgical peri-implantitis treatment has proven to be a challenge, with cumulative survival rates varying between 21.3 and 70.7% with a cumulative survival time of 5.67–9.95 years, depending on disease severity [21]. Long-term follow-up of the patients in this study should reveal if the achieved peri-implant status can be maintained.

Numerous treatment protocols exist for the surgical management of peri-implantitis, demonstrating a variety of decontamination techniques. However, no golden standard can yet be determined when it comes to the most effective treatment protocol to definitively suppress peri-implant inflammation in the long term [22]. Furthermore, decontamination protocols that incorporate implant surface modifying instruments, such as titanium brushes, could alter the implant surface in such a way that it compromises its biocompatibility [23]. The application of electrolytic cleaning does not seem to interfere with the implant surface integrity, which would hypothetically provide an optimal opportunity for re-osseointegration.

When closely inspecting the intraoral radiographs, it seems that the defect fill directly adjacent to the implant surface is progressively decreasing over time (or has not been accomplished at all, given that bone quality can only be assessed histologically; Figure 13, T_12_). Since all cases portray peri-implant BoP at T_12_, some level of peri-implant inflammation might still be present. It has been demonstrated that Gram-negative bacterial lipopolysaccharides (LPSs, endotoxins) adhere firmly to the implant surface and can maintain the peri-implant inflammation [24]. Although the colony forming units seem to decrease significantly after electrolytic cleaning (also when compared to other decontamination workflows), these endotoxins do not seem to be eliminated entirely [25]. Whether this LPS can serve as a substrate for the reemergence of peri-implant inflammation will require further investigation. However, it could explain why bone loss could reappear after 6 months of submerged healing.

Limitations of this study should be acknowledged. As a series of three cases, this design does not allow for definitive conclusions regarding treatment effectiveness, as would be possible in larger clinical studies. Given the case series nature involving three patients, the findings should be interpreted with caution. The limited sample size restricts both the efficacy of assessment and the generalizability of outcomes. Therefore, emphasizing the preliminary nature of these observations is important. Validation of the potential benefits of electrolytic cleaning in surgical peri-implantitis treatment requires controlled, prospective studies with larger populations, comparing electrolytic cleaning to, e.g., existing mechanical or chemical decontamination protocols. Furthermore, 3D radiographic imaging could have elevated diagnostics, providing a more precise assessment of preoperative peri-implant bone levels and the outcomes of site augmentation, which might offer valuable insights for future research. In addition, long-term evaluation remains crucial, as some parameters have not reached a stable level one year postoperatively. Therefore, extending the evaluation time could give more insights into the development of clinical radiographic parameters and the stability of peri-implant tissues. A follow-up period of at least three years following surgical intervention has been proposed as desirable to adequately evaluate the long-term stability of peri-implant tissues [26,27,28]. A key limitation of the current approach is that the use of the electrolytic cleaning device is restricted to cases in which the suprastructure can be removed. This prerequisite limits its applicability, particularly in situations involving cemented restorations or where prosthetic removal would compromise the integrity of the reconstruction. Furthermore, technical challenges may arise when the spray head is unable to make contact with the implant surface due to, e.g., limited interproximal space. To date, there is limited clinical evidence documenting adverse effects associated with the use of electrolytic cleaning in the surgical treatment of peri-implantitis. When it comes to implant surface alterations, some concerns are raised regarding the possibility of pitting corrosion, a localized form of corrosion that can lead to the formation of small holes or pits on the implant surface [29]. Also, the current body of evidence regarding electrolytic cleaning is predominantly focused on titanium implants, and due to the distinct electrochemical properties of zirconia, its applicability to zirconia implants remains uncertain and potentially limited.

In short, this case series provides a comprehensive overview of the use of electrolytic cleaning during surgical peri-implantitis treatment. The patients presented in this study posed challenging cases that showed several improvements both clinically and radiographically, one year after surgical peri-implantitis treatment.

## 9. Conclusions

Based on these cases, it can be concluded that electrolytic cleaning during the surgical treatment of peri-implantitis could be a promising addition to the available decontamination protocols. At T_12_, notable clinical and radiographical improvements can be observed, including reductions in both bleeding and suppuration scores as well as enhanced radiographic bone levels and probing depths. This should be further investigated in prospective studies comparing this decontamination technique to existing techniques. Future research could explore the potential synergistic effects of electrolytic cleaning therapy in combination with other adjunctive treatments. Investigating a combined approach could provide further insights into their mutual influence on peri-implant tissue healing and the overall effectiveness of peri-implantitis management strategies.

## Figures and Tables

**Table 1:** Patient characteristics.

	Case 1	Case 2	Case 3
Gender (female/male)	Female	Male	Female
Age at inclusion (years)	48	65	57
Implant diameter (mm)	3.3	3.5	4.1
Implant length (mm)	10.0	16.0	12.0
Implant position	22	12	21
Suprastructure	Screw-retained crown	Screw-retained crown	Screw-retained crown

**Table 2:** Clinical and radiographic outcomes.

	Case 1	Case 2	Case 3
Mean peri-implant pocket depth at T_pre_ (mm)	8.5	8.5	8.5
Mean peri-implant pocket depth at T_9_ (mm)	3.2	3.5	3.0
Mean peri-implant pocket depth at T_12_ (mm)	3.2	4.0	3.0
Deepest peri-implant pocket at T_pre_ (mm)	11.0	10.0	9.0
Deepest peri-implant pocket at T_9_ (mm)	4.0	6.0	4.0
Deepest peri-implant pocket at T_12_ (mm)	5.0	6.0	4.0
Peri-implant plaque at T_pre_ (%)	0.0	16.7	0.0
Peri-implant plaque at T_9_ (%)	0.0	66.7	0.0
Peri-implant plaque at T_12_ (%)	16.7	16.7	0.0
Peri-implant BS at T_pre_ (%)	100.0	100.0	100.0
Peri-implant BS at T_9_ (%)	100.0	33.3	16.7
Peri-implant BS at T_12_ (%)	16.7	83.3	33.3
Peri-implant SS at T_pre_ (%)	16.7	66.7	50.0
Peri-implant SS at T_9_ (%)	0.0	0.0	0.0
Peri-implant SS at T_12_ (%)	0.0	0.0	0.0
Midfacial mucosa level at T_pre_ (mm)	3.0	2.0	5.0
Midfacial mucosa level at T_9_ (mm)	5.0	6.0	6.0
Midfacial mucosa level at T_12_ (mm)	5.0	6.0	6.0
Midfacial keratinized tissue width at T_pre_ (mm)	3.0	7.0	5.0
Midfacial keratinized tissue width at T_9_ (mm)	3.0	3.0	3.0
Midfacial keratinized tissue width at T_12_ (mm)	3.0	3.0	2.0
Radiographic bone level at T_pre_ mesial (mm)	5.6	3.8	1.7
Radiographic bone level at T_pre_ distal (mm)	7.6	4.2	3.2
Radiographic bone level at T_2_ mesial (mm)	0.4	3.5	0.0
Radiographic bone level at T_2_ distal (mm)	1.8	3.9	0.0
Radiographic bone level at T_6_ mesial (mm)	2.8	3.4	1.1
Radiographic bone level at T_6_ distal (mm)	3.5	0.7	2.5
Radiographic bone level at T_12_ mesial (mm)	2.3	2.5	1.7
Radiographic bone level at T_12_ distal (mm)	3.5	1.5	2.5

**Table 3:** Pink Esthetic Score.

	Case 1		Case 2		Case 3	
	T_pre_	T_12_	T_pre_	T_12_	T_pre_	T_12_
Mesial papilla	1	1	0	0	2	2
Distal papilla	1	1	0	0	2	1
Curvature of the facial mucosa	1	1	2	2	1	2
Level of the facial mucosa	0	2	2	1	1	2
Root convexity/soft tissue color and texture	0	1	2	2	0	1
Total	3	6	6	5	6	8

## Data Availability

The original contributions presented in this study are included in the article. Further inquiries can be directed to the corresponding author.

## References

[1] Berglundh T., Armitage G., Araujo M.G., Avila-Ortiz G., Blanco J., Camargo P.M., Chen S., Cochran D., Derks J., Figuero E. (2018). Peri-implant diseases and conditions: Consensus report of workgroup 4 of the 2017 World Workshop on the Classification of Periodontal and Peri-Implant Diseases and Conditions. J. Clin. Periodontol..

[2] Jepsen S., Schwarz F., Cordaro L., Derks J., Hämmerle C.H.F., Heitz-Mayfield L.J., Hernández-Alfaro F., Meijer H.J.A., Naenni N., Ortiz-Vigón A. (2019). Regeneration of alveolar ridge defects. Consensus report of group 4 of the 15th European Workshop on Periodontology on Bone Regeneration. J. Clin. Periodontol..

[3] De Waal Y.C.M., Raghoebar G.M., Meijer H.J.A., Winkel E.G., Van Winkelhoff A.J. (2015). Implant decontamination with 2% chlorhexidine during surgical peri-implantitis treatment: A randomized, double-blind, controlled trial. Clin. Oral Implant. Res..

[4] Hentenaar D.F.M., De Waal Y.C.M., Stewart R.E., Van Winkelhoff A.J., Meijer H.J.A., Raghoebar G.M. (2022). Erythritol air polishing in the surgical treatment of peri-implantitis: A randomized controlled trial. Clin. Oral Implant. Res..

[5] Heitz-Mayfield L.J.A., Heitz F., Koong B., Huang T., Chivers P. (2023). Surgical peri-implantitis treatment with and without guided bone regeneration. A randomized controlled trial. Clin. Oral Implant. Res..

[6] Derks J., Ortiz-Vigón A., Guerrero A., Donati M., Bressan E., Ghensi P., Schaller D., Tomasi C., Karlsson K., Abrahamsson I. (2022). Reconstructive surgical therapy of peri-implantitis: A multicenter randomized controlled clinical trial. Clin. Oral Implant. Res..

[7] Donos N., Calciolari E., Ghuman M., Baccini M., Sousa V., Nibali L. (2023). The efficacy of bone reconstructive therapies in the management of peri-implantitis. A systematic review and meta-analysis. J. Clin. Periodontol..

[8] Sahrmann P., Ronay V., Hofer D., Attin T., Jung R.E., Schmidlin P.R. (2015). In vitro cleaning potential of three different implant debridement methods. Clin. Oral Implant. Res..

[9] Wei M.C.T., Tran C., Meredith N., Walsh L.J. (2017). Effectiveness of implant surface debridement using particle beams at differing air pressures. Clin. Exp. Dent. Res..

[10] Gosau M., Hahnel S., Schwarz F., Gerlach T., Reichert T.E., Bürgers R. (2010). Effect of six different peri-implantitis disinfection methods on in vivo human oral biofilm. Clin. Oral Implant. Res..

[11] Schlee M., Wang H., Stumpf T., Brodbeck U., Bosshardt D., Rathe F. (2021). Treatment of Periimplantitis with Electrolytic Cleaning versus Mechanical and Electrolytic Cleaning: 18-Month Results from a Randomized Controlled Clinical Trial. J. Clin. Med..

[12] Ratka C., Weigl P., Henrich D., Koch F., Schlee M., Zipprich H. (2019). The Effect of In Vitro Electrolytic Cleaning on Biofilm-Contaminated Implant Surfaces. J. Clin. Med..

[13] Assunção M.A., Botelho J., Machado V., Proença L., Matos A.P.A., Mendes J.J., Bessa L.J., Taveira N., Santos A. (2023). Dental Implant Surface Decontamination and Surface Change of an Electrolytic Method versus Mechanical Approaches: A Pilot In Vitro Study. J. Clin. Med..

[14] Monje A., Pons R., Peña P. (2025). Electrolytic Surface Decontamination in the Reconstructive Therapy of Peri-Implantitis: Single-Center Outcomes. Int. J. Periodontics Restor. Dent..

[15] Riley D.S., Barber M.S., Kienle G.S., Aronson J.K., von Schoen-Angerer T., Tugwell P., Kiene H., Helfand M., Altman D.G., Sox H. (2017). CARE guidelines for case reports: Explanation and elaboration document. J. Clin. Epidemiol..

[16] Belser U.C., Grütter L., Vailati F., Bornstein M.M., Weber H., Buser D. (2009). Outcome Evaluation of Early Placed Maxillary Anterior Single-Tooth Implants Using Objective Esthetic Criteria: A Cross-Sectional, Retrospective Study in 45 Patients With a 2- to 4-Year Follow-Up Using Pink and White Esthetic Scores. J. Periodontol..

[17] Tomasi C., Regidor E., Ortiz-Vigón A., Derks J. (2019). Efficacy of reconstructive surgical therapy at peri-implantitis-related bone defects. A systematic review and meta-analysis. J. Clin. Periodontol..

[18] Aghazadeh A., Persson R.G., Renvert S. (2020). Impact of bone defect morphology on the outcome of reconstructive treatment of peri-implantitis. Int. J. Implant. Dent..

[19] Monje A., Pons R., Sculean A., Nart J., Wang H. (2023). Defect angle as prognostic indicator in the reconstructive therapy of peri-implantitis. Clin. Implant. Dent. Relat. Res..

[20] Herrera D., Berglundh T., Schwarz F., Chapple I., Jepsen S., Sculean A., Kebschull M., Papapanou P.N., Tonetti M.S., Sanz M. (2023). Prevention and treatment of peri-implant diseases-The EFP S3 level clinical practice guideline. J. Clin. Periodontol..

[21] Hwang S., Lee H., Yun P., Kim Y. (2023). Survival analysis of implants after surgical treatment of peri-implantitis based on bone loss severity and surgical technique: A retrospective study. BMC Oral Health.

[22] Baima G., Citterio F., Romandini M., Romano F., Mariani G.M., Buduneli N., Aimetti M. (2022). Surface decontamination protocols for surgical treatment of peri-implantitis: A systematic review with meta-analysis. Clin. Oral Implant. Res..

[23] Louropoulou A., Slot D.E., Van der Weijden F. (2014). The effects of mechanical instruments on contaminated titanium dental implant surfaces: A systematic review. Clin. Oral Implant. Res..

[24] Barão V.A., Mathew M.T., Assunção W.G., Yuan J.C., Wimmer M.A., Sukotjo C. (2011). The role of lipopolysaccharide on the electrochemical behavior of titanium. J. Dent. Res..

[25] Zhu Y., Xu Y., Ling Z., Zhao C., Xu A., He F. (2024). The biofilm removal effect and osteogenic potential on the titanium surface by electrolytic cleaning: An in vitro comparison of electrolytic parameters and five techniques. Clin. Oral Implant. Res..

[26] Carcuac O., Derks J., Abrahamsson I., Wennström J.L., Berglundh T. (2020). Risk for recurrence of disease following surgical therapy of peri-implantitis-A prospective longitudinal study. Clin. Oral Implant. Res..

[27] Lee S., Lee B., Choi S., Kim Y. (2021). Long-term outcomes after peri-implantitis treatment and their influencing factors: A retrospective study. J. Periodontal Implant. Sci..

[28] Serino G., Wada M., Mameno T., Renvert S. (2021). Two- and ten-year follow-up of patients responding and non-responding to the surgical treatment of peri-implantitis: A retrospective evaluation. Clin. Oral Implant. Res..

[29] Kaiser F., Schwarnweber D., Bierbaum S., Wolf-Brandstetter C. (2020). Success and side effects of different treatment options in the low current attack of bacterial biofilms on titanium implants. Bioelectrochemistry.

